# Between the lines: investigating health beliefs and emotional expressions in online mental health communities

**DOI:** 10.3389/fpsyg.2025.1521623

**Published:** 2025-10-24

**Authors:** Khanh Nguyen, Binh Vu, Swati Chandna, Jobst-Hendrik Schultz, Gwendolyn Mayer

**Affiliations:** ^1^Department of Applied Data Science and Analytics, SRH University Heidelberg, Heidelberg, Germany; ^2^Department of General Internal Medicine and Psychosomatics, Heidelberg University Hospital, Heidelberg, Germany

**Keywords:** mental health, depression, anxiety, natural language processing, Reddit, health belief model, sentiment (SEN) analysis, emotions

## Abstract

**Introduction:**

Social media platforms play an important role in mental health discourse. Applying the Health Belief Model (HBM) to health-related discussions on Reddit could yield deeper insights into individuals' perceptions of mental health threats and barriers to seeking help. The primary objective of this research is to develop an efficient methodology not only for classifying key HBM components—such as perceived susceptibility, severity, benefits, barriers, cues to action, and self-efficacy—but also for examining emotional expressions within these discussions.

**Methods:**

A sample of 5,000 posts was selected for classification and a subset was manually labelled for further analysis. Multiple models were tested in classification tasks. Data analysis utilized visualization techniques—such as word clouds, heatmaps, and emotional content analysis—to identify thematic trends and emotional expressions in the discussions.

**Results:**

DistilBERT outperformed other approaches, achieving accuracy rates between 75 and 84% for most components. However, challenges persist in predicting perceived severity, with an accuracy of only 47% due to its multi-label nature; to address this, GPT-4-based keyword extraction was combined with human review, improving accuracy to 81%. The emotional content analysis reveals patterns in mental health discussions, such as the attribution of personality as a root cause of anxiety by users and the urgent need for targeted interventions in cases of suicidal ideation.

**Discussion:**

Findings demonstrate that users tend to use more negative language in contexts with higher perceived severity. Future work should prioritize improving model adaptability to health-specific data, handling rare terms, conducting nuanced emotional analyses in written expressions, and addressing ethical implications in analyzing user-generated content.

## 1 Introduction

### 1.1 Background and related work

**Figure 1:**
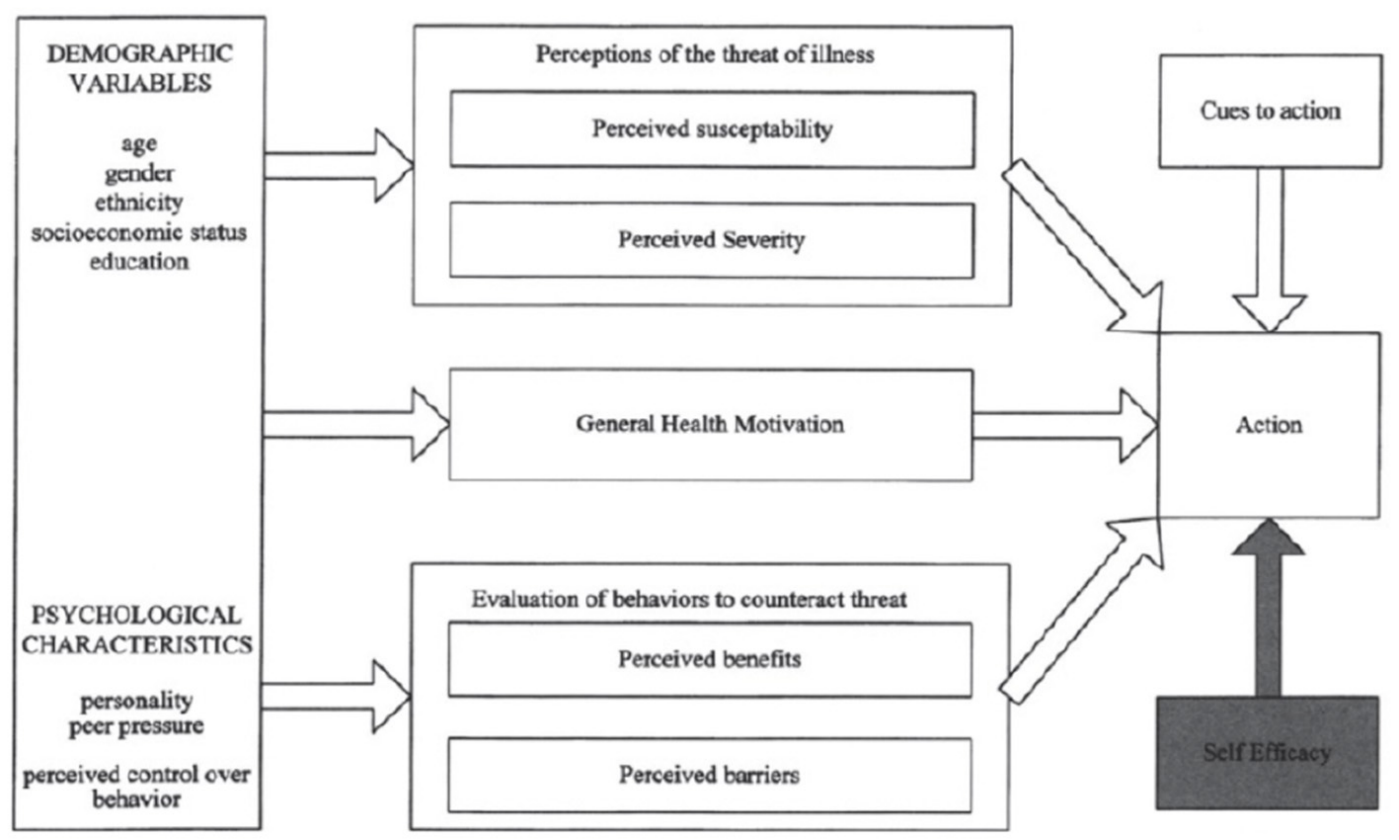
The modified health belief model by Rosenstock et al. (1988).

The HBM has been instrumental in understanding health behaviors since its inception in the 1950s, originally developed to explain the low uptake of tuberculosis screenings (Rosenstock, 1974). HBM incorporates key constructs such as perceived susceptibility, severity, benefits, barriers, and cues to action, later expanded in 1988 to include “self-efficacy” to better predict sustained behavior change (Rosenstock et al., 1988) (see Figure 1).

The HBM is essential for understanding health-related behaviors, such as in vaccine acceptance. Jones et al. (2015) highlighted the HBM's effectiveness as an explanatory framework in communication research, showcasing its ability to elucidate various mediation effects on health behaviors. This relevance is further demonstrated by Wong et al. (2021), who applied the HBM to assess COVID-19 vaccine acceptance in Hong Kong. Their findings indicated that perceived benefits, cues to action, and self-efficacy were significant predictors of vaccine uptake, while perceived severity and susceptibility had lesser impacts. These studies underscore the HBM's practical utility in shaping public health interventions by identifying key beliefs that influence health decisions, ultimately aiding in the development of targeted strategies to enhance vaccine acceptance and improve public health outcomes (Jones et al., 2015; Wong et al., 2021).

Mental health remains essential for individual wellbeing, allowing people to meet life's demands, realize their potential, and meaningfully participate in society. Despite numerous treatment options, the mental health care system often fails to meet population needs due to gaps in mental health policy, resource limitations, and overburdened healthcare systems, resulting in extended wait times and inconsistent care (Moitra et al., 2022). In 2019, around 970 million people experienced mental health issues worldwide, which, beyond individual suffering, impacted relationships, education, and employment while contributing to economic losses through reduced productivity (World Health Organization, 2019). Social stigma adds to these challenges, particularly affecting open discussions and help-seeking behavior. Social media platforms, notably Reddit, Twitter, and Instagram, now play an important role in mental health discourse. These platforms allow users to discuss mental health experiences, seek support, confront and transform stigmas openly. However, analyzing large datasets from social media poses challenges due to high volume and complexity. Traditional qualitative methods are valuable for accuracy but are often impractical at scale, prompting a shift toward advanced natural language processing (NLP) and machine learning methods that can automate such analyses.

Advances in large language models (LLMs) enable text processing with unprecedented precision, particularly useful for mental health analysis across extensive datasets. Earlier text representation models, such as Bag of Words (BoW) and n-grams, lacked semantic depth, but subsequent models like Word2Vec, Global Vectors for Word Representation (GloVe), and FastText improved text analysis by capturing nuanced semantic relationships and subword information (Mikolov et al., 2013; Pennington et al., 2014). Transformer-based architectures, notably Bidirectional Encoder Representations from Transformers (BERT) and Generative Pre-trained Transformer (GPT), introduced self-attention mechanisms that allow for the effective modeling of long-range dependencies, setting new standards for performance in NLP tasks (Vaswani et al., 2017; Devlin et al., 2018). Generative AI models like Meta's LLAMA3 and OpenAI's GPT-4 have further advanced NLP capabilities, particularly in tasks requiring complex text generation, contextual understanding, and multimodal reasoning. These models facilitate a range of applications, from emotional analysis to detailed text generation, effectively leveraging diverse online datasets for in-depth analysis of health discourse (Touvron et al., 2023; OpenAI, 2023).

Preprocessing and training strategies are crucial for optimizing classification models like DistilBERT and Robustly Optimized BERT Approach (RoBERTa) when analyzing user-generated content from platforms like Reddit (Sanh et al., 2019; Liu et al., 2019). Essential steps include text normalization, accent stripping, and special character removal, which improve model consistency and accuracy in emotional and belief-based analyses (Uysal and Gunal, 2014; Denny and Spirling, 2017). Data scarcity and class imbalance present additional challenges, particularly for detecting health beliefs in Reddit posts; data augmentation techniques, including contextual embeddings and generative models, help provide diverse samples, bolstering model robustness (Liu et al., 2019).

Rani et al. (2024) provided a valuable annotation of a rich Reddit dataset from the pandemic era, focusing on techniques to categorize root causes of mental health issues. However, the paper primarily emphasizes methodological aspects, leaving an opportunity for further analysis. Applying HBM to this dataset could yield deeper insights into individuals' perceptions of mental health threats and barriers to seeking help, thereby informing more targeted interventions and support strategies. Existing literature highlights the need for accurate HBM classification in social media health research, with studies emphasizing both manual labeling for reliability and machine learning for scalability. Manual labeling is effective but can be time-intensive, as noted by Jones et al. (2015). Machine learning, while powerful, relies heavily on high-quality training data and may miss contextual nuances without sufficient human input (Shorten et al., 2021). Hybrid approaches, blending rule-based systems and active learning, offer a balanced solution, though they are complex and resource-intensive (Sahin et al., 2012). Emotion classification methods like Text2emotion and NRCLex use fixed lexicons, while ML-based tools, such as IBM's Tone Analyzer, offer higher contextual accuracy at increased computational costs (Cambria, 2016). Studies like Du et al. (2019) demonstrate the feasibility of classifying HBM constructs using deep learning but encounter limitations in addressing platform-specific language evolution.

### 1.2 Objective

The objective of this research is to develop a computational methodology for analyzing Reddit data through the lens of the Health Belief Model, aiming to understand how health beliefs and emotional content shape public discourse on mental health. Key research questions focus on identifying effective NLP and ML techniques for accurately categorizing health beliefs and emotions in mental health discussions, as well as examining the interaction between emotional expression and health beliefs to better understand public engagement with mental health topics online. This research seeks to combine manual and automated analysis techniques to ensure scalable, contextually nuanced insights that contribute to both academic research and practical applications in health communication strategies.

## 2 Dataset and methodology

### 2.1 Dataset

The raw dataset was sourced from Reddit by Rani et al. (2024) and encompasses posts from five subreddits: anxiety, loneliness, mental health, suicide watch, and depression. Collected in 2022, it includes posts spanning from 2019 to 2022. The original study aimed to explore perceived causes of mental health issues through an analysis of 800 expert-annotated posts.

The dataset consists of millions of rows and seven columns, incorporating both qualitative features (Title, Author, Selftext, Subreddit) and quantitative features (Score, Created_utc, Timestamp). Among these, the labeled dataset contains 800 entries with two relevant columns: Label, representing the root cause, and CAT1, providing a deeper, more detailed level of the root cause, as illustrated in Table 1 (Rani et al., 2024). While CAT1 offers additional granularity, this research focuses primarily on the Health Belief Model, so that column was not used in the analysis. This research specifically analyzes a subset of posts from May to July 2022, following the onset of the pandemic.

**Table 1:** Data types of labeled dataset.

**Number**	**Column**	**Non-null count**	**Dtype**
1	Score	800	Int64
2	Selftext	800	Object
3	Subreddit	800	Object
4	Title	800	Object
5	Label (root cause)	800	Object
6	CAT 1	200	Object

#### 2.1.1 Ethical considerations

No Ethical approval was obtained for the purpose of this study. The original dataset was analyzed in accordance with the local Ethics Committee of Victoria University, Melbourne (Rani et al., 2024). Additional Ethical measures were taken, to protect user privacy: All columns containing usernames or other identifiers were removed, and posts that included personal information were excluded.

### 2.2 Methodology

#### 2.2.1 Sample selection

To analyze 1 million Reddit posts, a strategic sampling approach was employed that balances statistical validity with practical constraints.

1. Overall sample: 5,000 posts were selected, sufficient for stable results in large corpora analysis (Qiu et al., 2014).
2. Manual labeling for training: a range of 500–750 posts was used to ensure adequate representation for each class, adhering to the guideline of at least 100 samples per class Beleites et al. (2013).
3. Testing sample: 125–225 posts were allocated, maintaining a typical 70/30 to 80/20 training/testing split in machine learning.
4. Validation of machine-labeled data: the validation requires 288–300 posts, adjusting for a 95% confidence level and a 5.7% margin of error, determined using Cochran (1977).

#### 2.2.2 Analytical process flow

**Figure 2:**
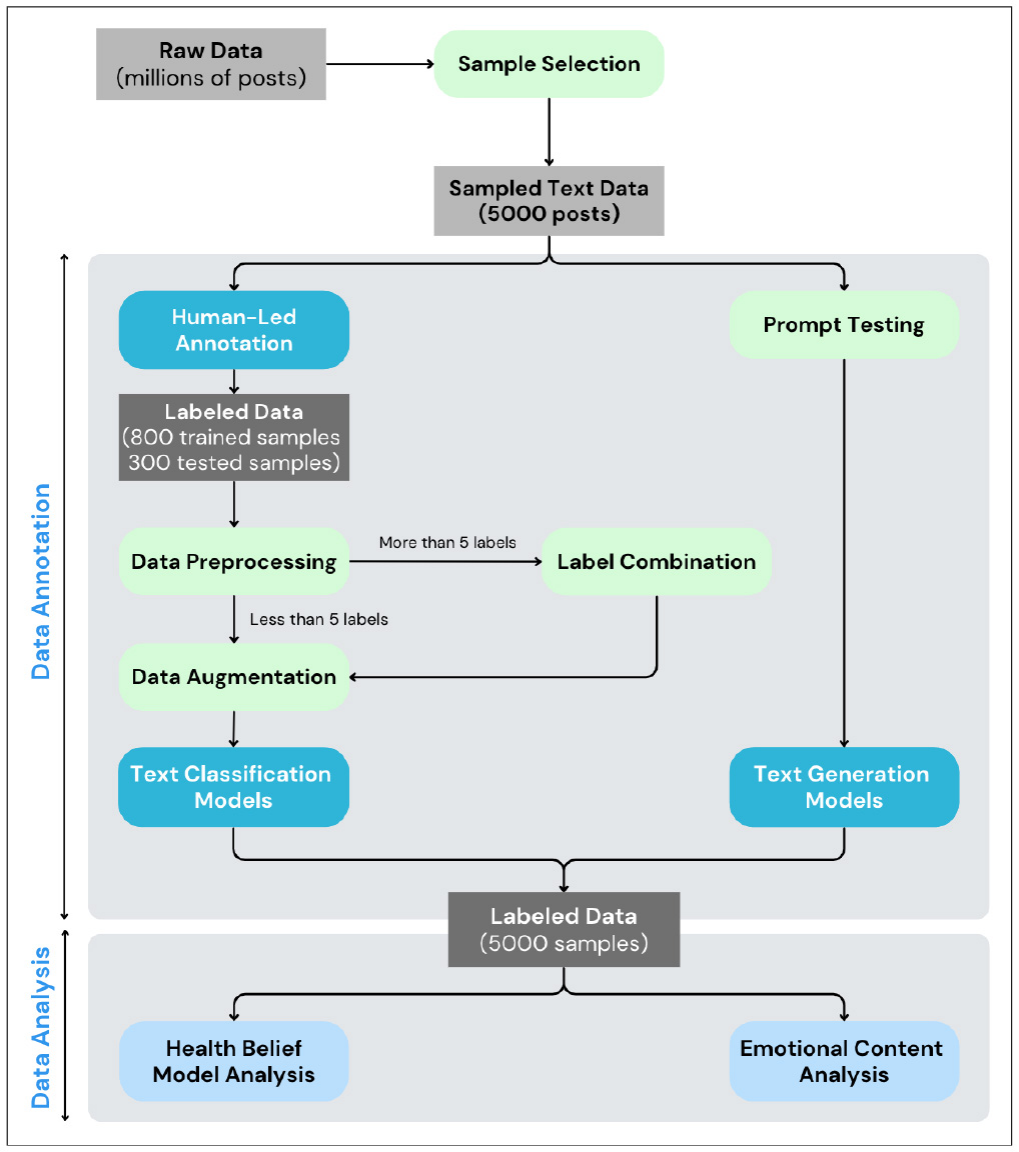
Overall flow from data annotation to data analysis.

The analytical process flow is shown in Figure 2. From the original dataset, 5,000 posts were randomly selected for analysis. Two annotators labeled the posts based on Health Belief Model dimensions, including perceived susceptibility, severity, benefits, barriers, cues to action, and self-efficacy. This labeled data was then combined with 800 labeled rows identifying root causes of mental health issues from a previous study by Rani et al. (2024) to train the model. Based on the outcomes, 288–300 posts were chosen for further evaluation, aiming for an accuracy rate above 75% before examining the HBM and emotional content.

#### 2.2.3 Data annotation

Human-led annotation was vital for preparing the dataset. Two annotators, from the Data and Psychology teams, labeled the posts based on the Health Belief Model dimensions. Initial discrepancies were resolved through discussion to ensure consistent labeling.

To ensure adequate context for labeling, posts with fewer than 50 characters were excluded. The preprocessing phase ensured dataset consistency and readiness for analysis, focusing on handling missing values, removing duplicates, and normalizing text. These steps prepared the data for effective tokenization and model training. Tokenization involved converting text into a machine-readable format, using advanced tokenizers compatible with models like RoBERTa and DistilBERT. Texts were truncated to meet input length constraints, ensuring efficient data handling and model performance.

To address class imbalance, labels making up less than 7% of the dataset were consolidated into an “others” category, enhancing model accuracy. Furthermore, the use of data augmentation techniques, specifically through the “nlpaug.augmentation” package, facilitated the generation of synthetic samples, thereby enhancing the diversity and robustness of the dataset.

The dataset was split into training and testing subsets at an 80/20 ratio, allowing for substantial training while retaining data for unbiased evaluation. The training and testing processes were conducted in a GPU-enabled cloud-based environment, providing the necessary computational power to handle large datasets and optimize model performance effectively. Model performance was assessed using accuracy, precision, recall, and F1-score, providing insights into their effectiveness.

Regarding data annotation using the GPT model, multiple prompts were iteratively tested and refined. The final prompt presented below was selected to classify the root causes and components of HBM.

*You are an experienced psychiatrist. Analyze the following text based on the Health Belief Model. Categorize it into these seven categories:*.

. *Root cause: root cause of mental health problems (drug and alcohol, trauma and stress, personality, early life)*.

. *Sentiment: overall sentiment (positive, neutral, or negative)*.

. *Perceived severity: health issue or concern perceived as severe (depression, anxiety, suidecide attempt, hallucination, etc.)*

. *Perceived benefits: benefits perceived in taking health-seeking action (not mentioned, finding support, feeling heard and understood, getting access to treatment, etc.)*.

. *Perceived barriers: barriers perceived in taking health-seeking action (not mentioned, feeling helpless, feeling unheard and misunderstood, Lack of resources, etc.)*.

. *Cue to action: actions or reasons for taking steps to improve mental wellbeing (no action, sharing their situation, seeking for support and treatment, looking for resources, etc.)*

. *Self-efficacy: mindset (empowered, overcome, denial, trouble)*.

*Respond with a valid JSON object containing these seven categories as keys and your analysis as values. Keep each value concise, preferably not more than five words*.

To account for the range of mental health perceived severity an individual may experience, the objective was to ensure the text was as comprehensive as possible. Consequently, multiple labels were included despite potential challenges in training a model for perceived severity classification. A large number of labels and data imbalance can introduce difficulties in achieving accurate classification model training. Since the GPT model categorized perceived severity as high, medium, or low, the term “mental health issues” was accompanied by examples to enhance the results. This was implemented using the prompt below.

*You are an experienced psychiatrist. Analyze the following text:*

. *Health issue: Which mental health issue is mentioned in the text? Answers could be: depression, anxiety, suicide attempt, hallucinations, suicidal thoughts, loneliness, panic attacks, alcoholism, drug addiction, bipolar, trauma, stress, etc*.

. *Keywords: Keywords from text that relates to mental health issues*.

*Respond with a valid JSON object containing Health issue and Keywords as keys and your analysis as values. Keep each value concise, preferably not more than five words*.

#### 2.2.4 Data analysis

After labeling the data, various visualizations were employed to analyze the components of the Health Belief Model. For columns with multiple labels, such as perceived severity, a word cloud visualized the distribution of key terms. Correlation analysis was conducted to examine relationships, such as between sentiment scores and self-efficacy levels. This analysis assessed whether individuals with high perceived benefits reported lower barriers and how cues to action relate to other factors, exploring their roles as mediators or moderators in belief formation or behavior change.

To analyze the emotional content, a sentiment analysis was conducted for each word across different levels of perceived severity to assess whether users used more negative language in specific instances. Furthermore, the top 10 negative words in each group were identified to highlight distinct language patterns.

## 3 Results

### 3.1 Data annotation

#### 3.1.1 Human-lED sample annotation

**Figure 3:**
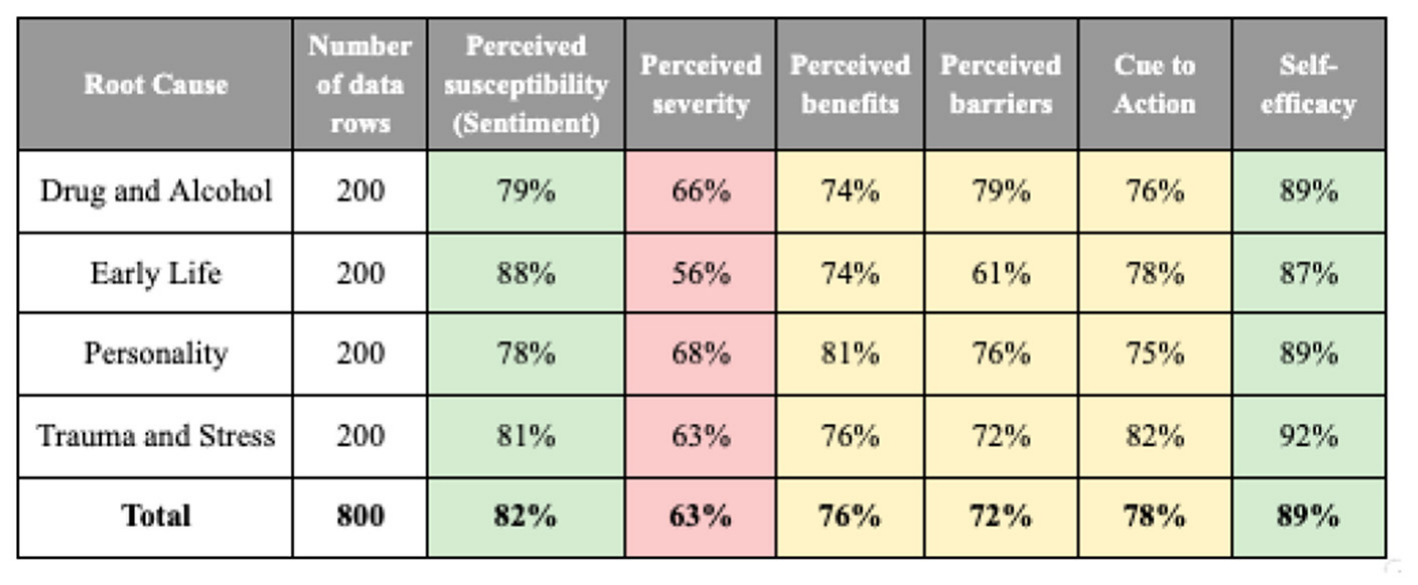
The initial results of a comparison between the annotations of two annotators (green = high, yellow = medium, red = low agreement).

Figure 3 summarizes the initial results comparing annotations from two annotators on various health belief components.

The results indicate that perceived susceptibility (81.5%) and self-efficacy (89.25%) had the highest alignment, while perceived severity (63.25%) exhibited the lowest agreement, due to the presence of multiple labels per text. This suggests that annotators more consistently agree on susceptibility and self-efficacy, whereas severity is more subjective and may require additional clarification or multiple indicators in the analysis.

**Figure 4:**
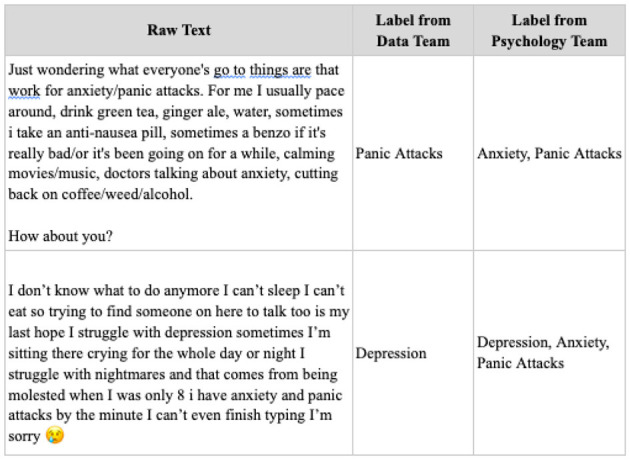
The sample labels from the two annotators.

Figure 4 shows discrepancies in the labeling of raw text by the data and psychology teams. These differences highlight the complexity of the task and indicate why training models—especially for accurately classifying perceived severity—can be challenging.

#### 3.1.2 Text classification models

The initial testing of various models revealed that DistilBERT, particularly when combined with data preprocessing and augmentation techniques, achieved the highest accuracy rates—86% for root cause classification and 89% for self-efficacy.

**Figure 5:**
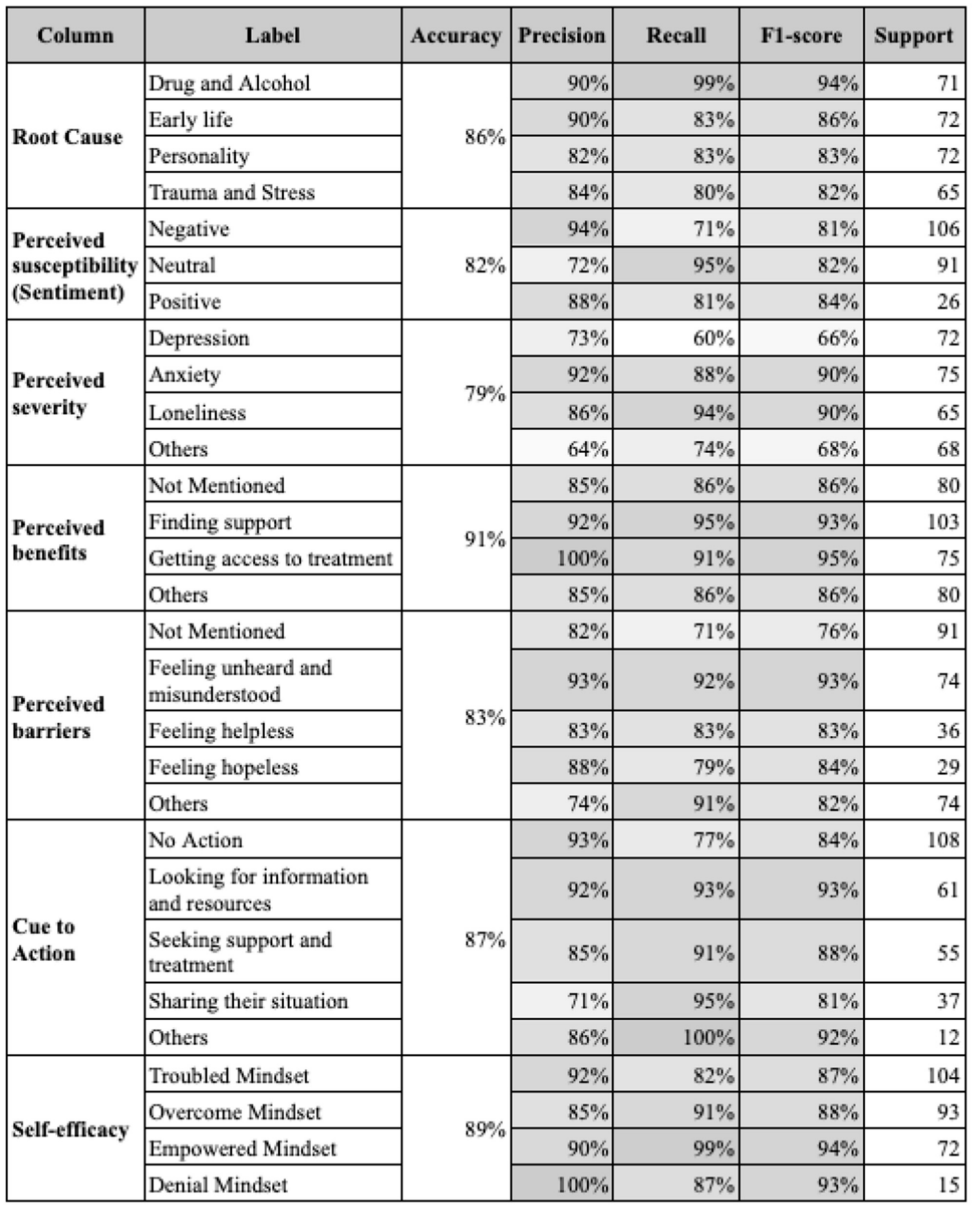
The performance metrics of final models for each column.

Figure 5 showcases the final performance metrics of models, revealing an overall precision exceeding 79% and F1 scores around 80%–90%. While most categories performed well, perceived severity had a notably lower accuracy of 47%, attributed to its inherent complexity and multi-label nature.

#### 3.1.3 Text generation models

**Figure 6:**
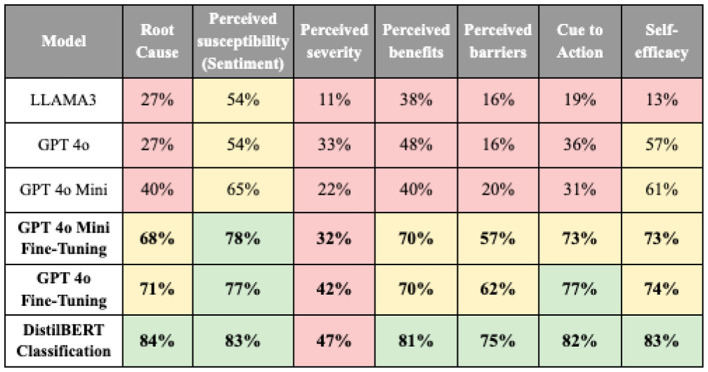
The performance metrics of final models for each column (green = high, yellow = medium, red = low performance).

Evaluation of the text generation models revealed that fine-tuned GPT-4 models (GPT4o and GPT4o mini) achieved accuracy rates between 62 and 77%, as shown in Figure 6. However, despite these solid results, the GPT-4 models consistently trailed behind DistilBERT classification in accuracy across all categories. Perceived severity had the lowest accuracy across all model predictions, primarily due to the complexity of managing multiple labels.

**Figure 7:**
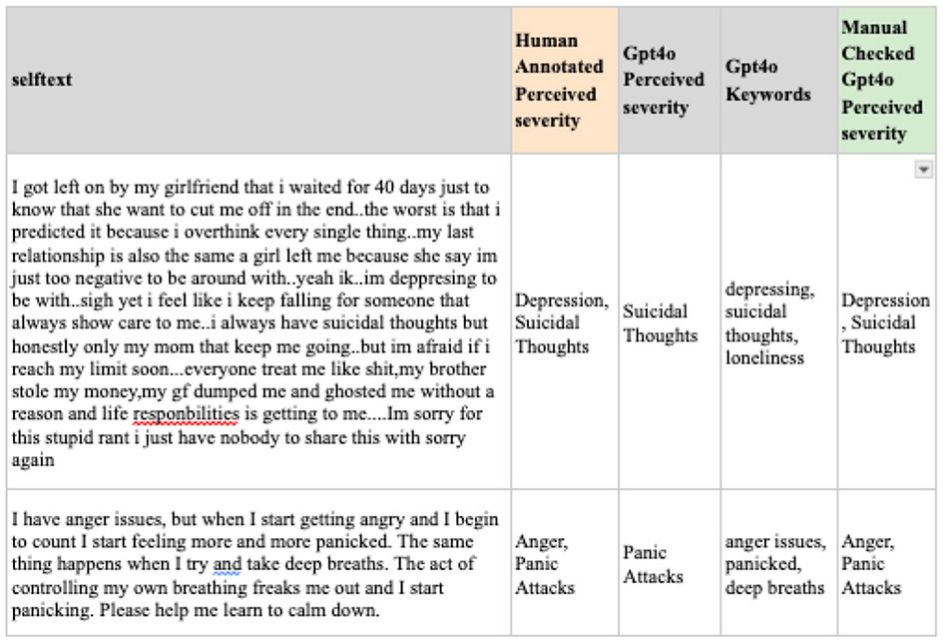
Examples of the predicted perceived severity after manual review.

To address this, predicting labels, extracting keywords related to mental health, and combining them for the final prediction is suggested. While the initial output from GPT model produced a single result for perceived severity, the subsequent extraction of keywords enabled a more precise identification of multiple severity-related dimensions. Figure 7 presents examples of the predicted perceived severity after manual review, including the perceived severity labels and keywords generated by GPT-4o. As illustrated, irrelevant terms (e.g., deep breaths) were excluded, whereas salient issues—such as anger and panic attacks-were retained. Although this approach requires additional manual verification, it achieves an accuracy rate of 81% and enhances data relevance for analysis.

### 3.2 Data analysis

#### 3.2.1 Health belief model analysis

The Health Belief Model Analysis examines how perceived severity, susceptibility, and self-efficacy shape health behaviors and perceptions in mental health contexts.

Regarding root cause, 50.1% of Reddit users attributed mental health problems to personality, with traits like chronic negativity or perfectionism increasing vulnerability. Trauma and stress accounted for 27.4%, followed by early life experiences (13.5%) and substance use (9.0%). In terms of perceived severity, depression and anxiety were the most discussed issues, with 1,471 and 1,415 posts, respectively, out of 5,000 analyzed.

**Figure 8:**
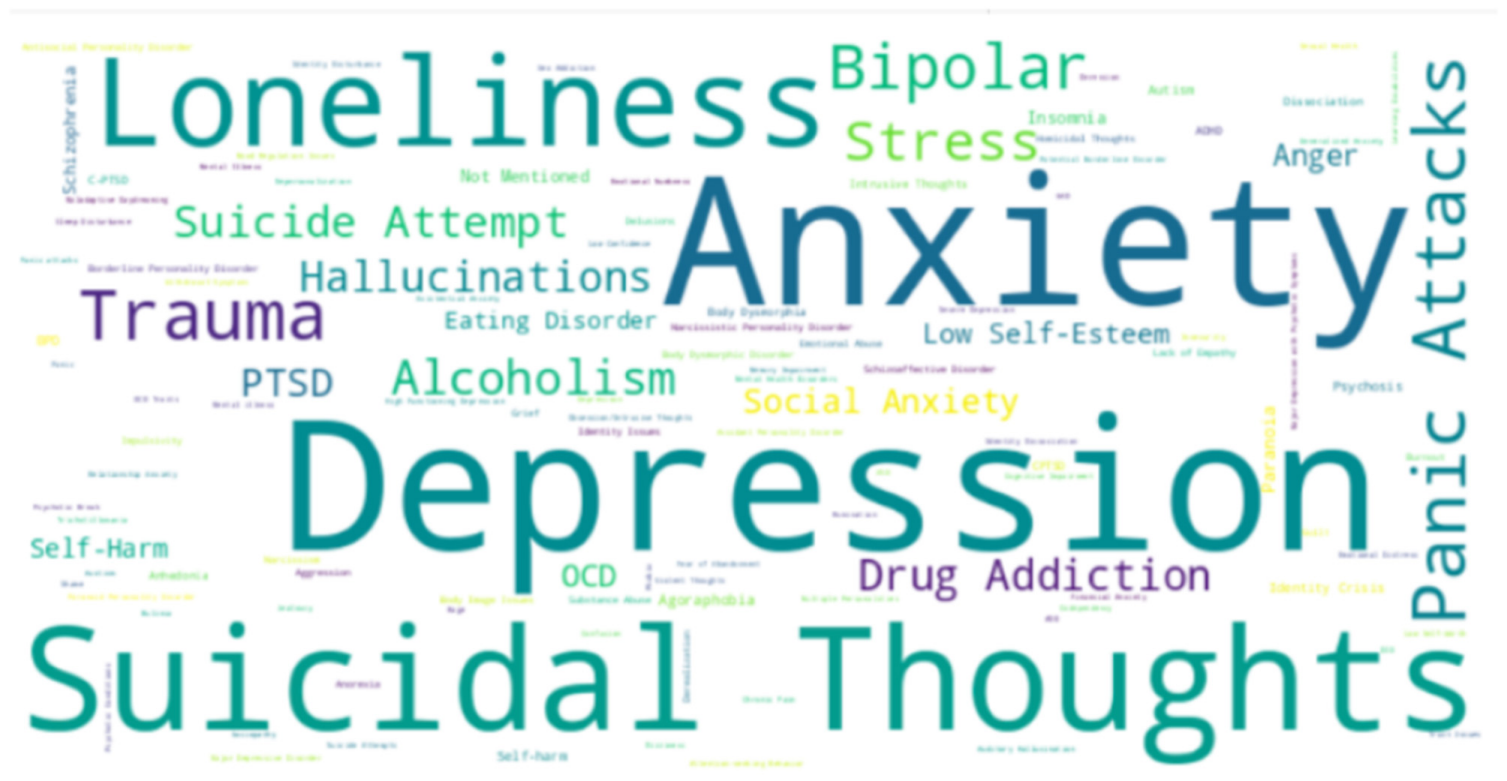
Perceived severity of whole dataset.

Other concerns included suicidal thoughts (1,125), loneliness (799), and panic attacks (150). Each post was counted individually, even when multiple issues were mentioned. Upon closer examination, distinct patterns emerged among the root causes. Anxiety was most frequently linked to personality (865 posts) and drug/alcohol use (164 posts). Loneliness was primarily linked to trauma and stress (485 posts), while depression was associated with both trauma and stress (341 posts) and early life experiences (217 posts) (see Figure 8).

**Figure 9:**
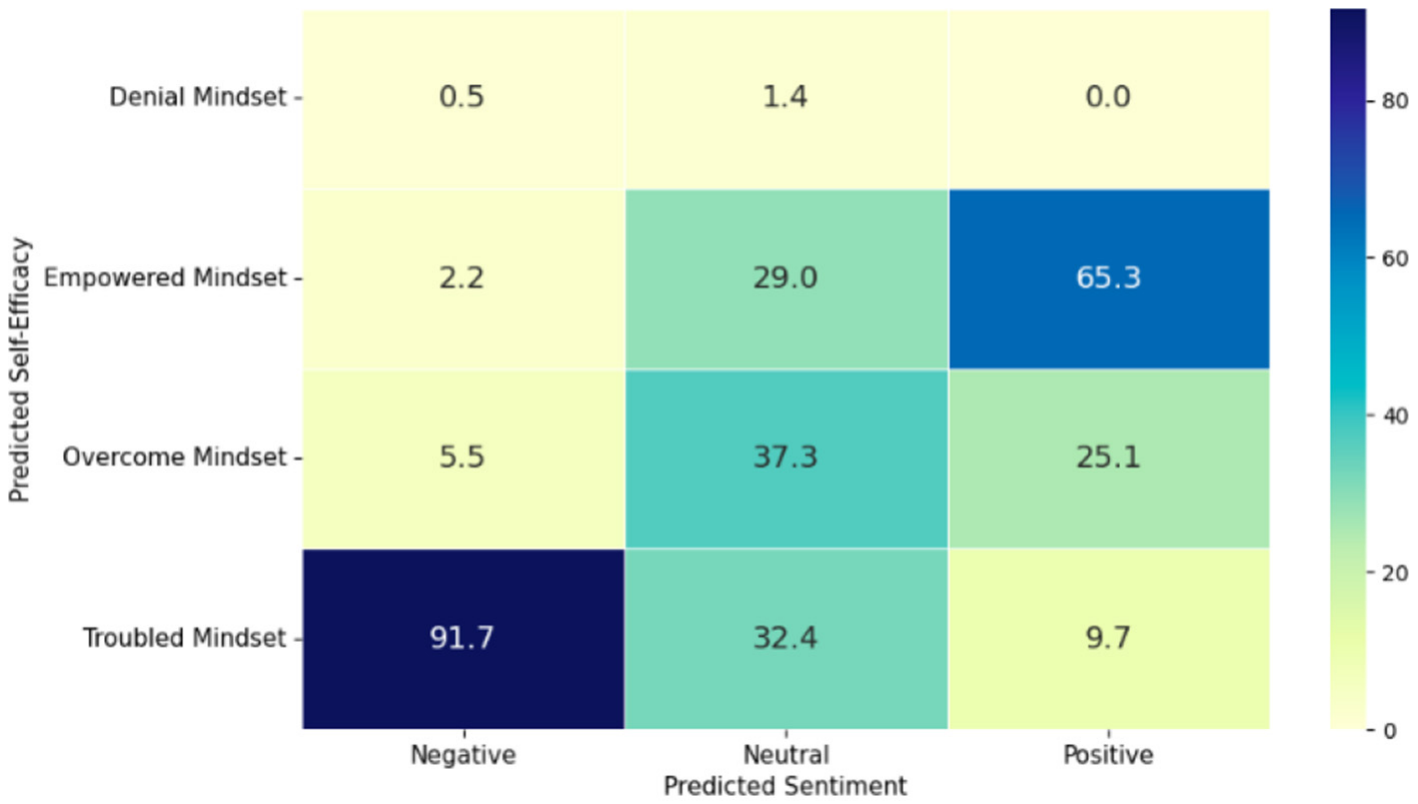
Percentages by predicted sentiment and self-efficacy.

Most perceptions about mental health were neutral (48.3%), with negative perceptions at 45.1% and only 6.6% expressing a positive outlook. A majority (57.6%) felt troubled in managing their mental health, while 22.2% felt they had overcome obstacles. A notable correlation existed between perceived susceptibility and self-efficacy; positive sentiments correlated with higher self-efficacy, whereas negative sentiments linked closely to a troubled mindset (see Figure 9).

Many respondents did not mention perceived benefits (69.0%) or barriers (67.3%). Most reported no action taken (63.1%). Among those who did identify benefits, finding support (21.0%) and accessing treatment (5.4%) were noted. Common barriers included feeling unheard (11.5%) and helpless (4.5%). In terms of actions taken, seeking information and support (16.3 and 12.1%, respectively) were most frequent.

Analysis across top perceived severity is described below:

Anxiety: personality (65.0%) was the primary root cause, with 70.8% of posts neutral in sentiment. Perceived benefits and barriers were largely unmentioned, with 31.1% seeking resources.
Depression: also primarily linked to personality (55.0%). Negative sentiment dominated (54.6%), with significant inaction noted (69.3%).
Anxiety and depression: similar trends to anxiety, indicating proactive behaviors despite challenges.
Suicidal thoughts: rooted in personality and trauma, these individuals faced overwhelmingly negative sentiments and high levels of inaction (82.2%).
Loneliness: often linked to trauma (61.5%), this group exhibited low engagement and high inaction.
Trauma or stress: root causes included personality (45.2%) and early life experiences (29.0%), with a mix of neutral and negative sentiments.
Alcoholism or drug addiction: predominantly driven by substance use (73.3%), individuals showed low self-efficacy and significant unreported barriers.
Others: various conditions showed personality as a main root cause, with many feeling troubled and inactive.

In summary, the analysis of the health belief model reveals that personality-related issues are common across different mental health conditions, but each issue has unique characteristics. Anxiety and depression underscore the importance of proactive interventions, while individuals dealing with suicidal thoughts, loneliness, and substance use challenges need focused support to effectively address their vulnerabilities.

#### 3.2.2 Emotional content analysis

Regarding word sentiment, the analysis shows that neutral sentiment dominated across all groups, comprising 89%–91%. Positive and negative sentiments varied among groups. Notably, only the loneliness group had a higher percentage of positive words (56.2%) than negative words (43.8%). In contrast, the groups with the highest negative word proportions were suicidal thoughts (58.1%), anxiety (57.1%), and others (56.4%).

**Figure 10:**
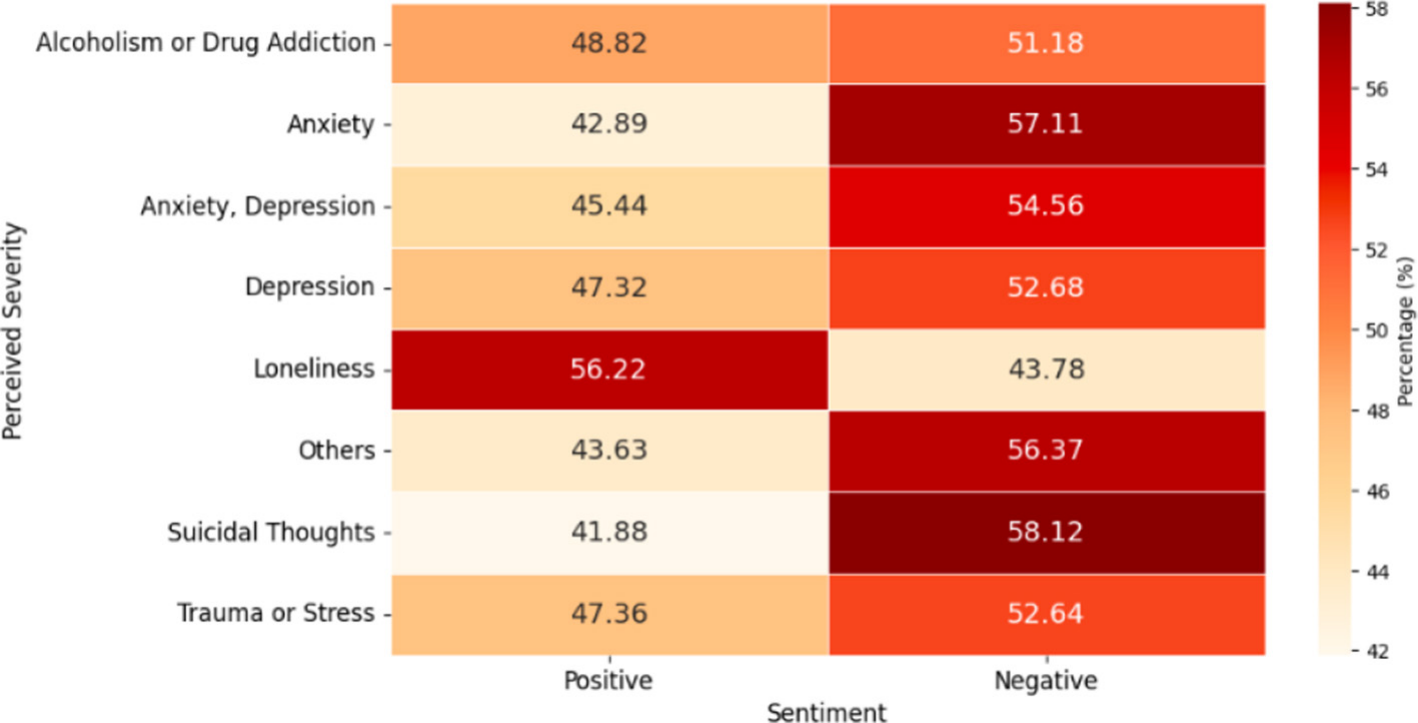
Percentages of word sentiment by predicted severity (excluding neutral).

Other groups exhibited negative word percentages between 51 and 55%, indicating that while loneliness had a more positive outlook, other issues were predominantly negative (see Figure 10).

This analysis identifies the top 10 negative words in each severity group, revealing emotional challenges specific to each mental health issue. The words “no” and “bad” consistently ranked among the top across all groups, with “no” being the top negative word in depression, others, loneliness, alcoholism or drug addiction, and trauma or stress categories.

For anxiety, prominent negative words included “anxiety” “anxious” “stop” and “worried” In depression, the most common terms were “depression” “hate” “lose” and “fuck” Suicidal thoughts featured words like “die” “kill” “suicidal” and “suicide.” In the Loneliness group, key words were “alone,” “lonely,” “hard” and “hate,” while in alcoholism or drug addiction, “stop” and “pain” were prevalent. The trauma or stress group highlighted words such as “stress” “trauma” and “stop” The “others” category featured “anxiety” “hard” “stop” and “hate.” Thus, while “no” and “bad” were common across all groups, each had unique words reflecting their emotional struggles.

#### 3.2.3 Summary of health belief model and emotional content analysis

**Figure 11:**
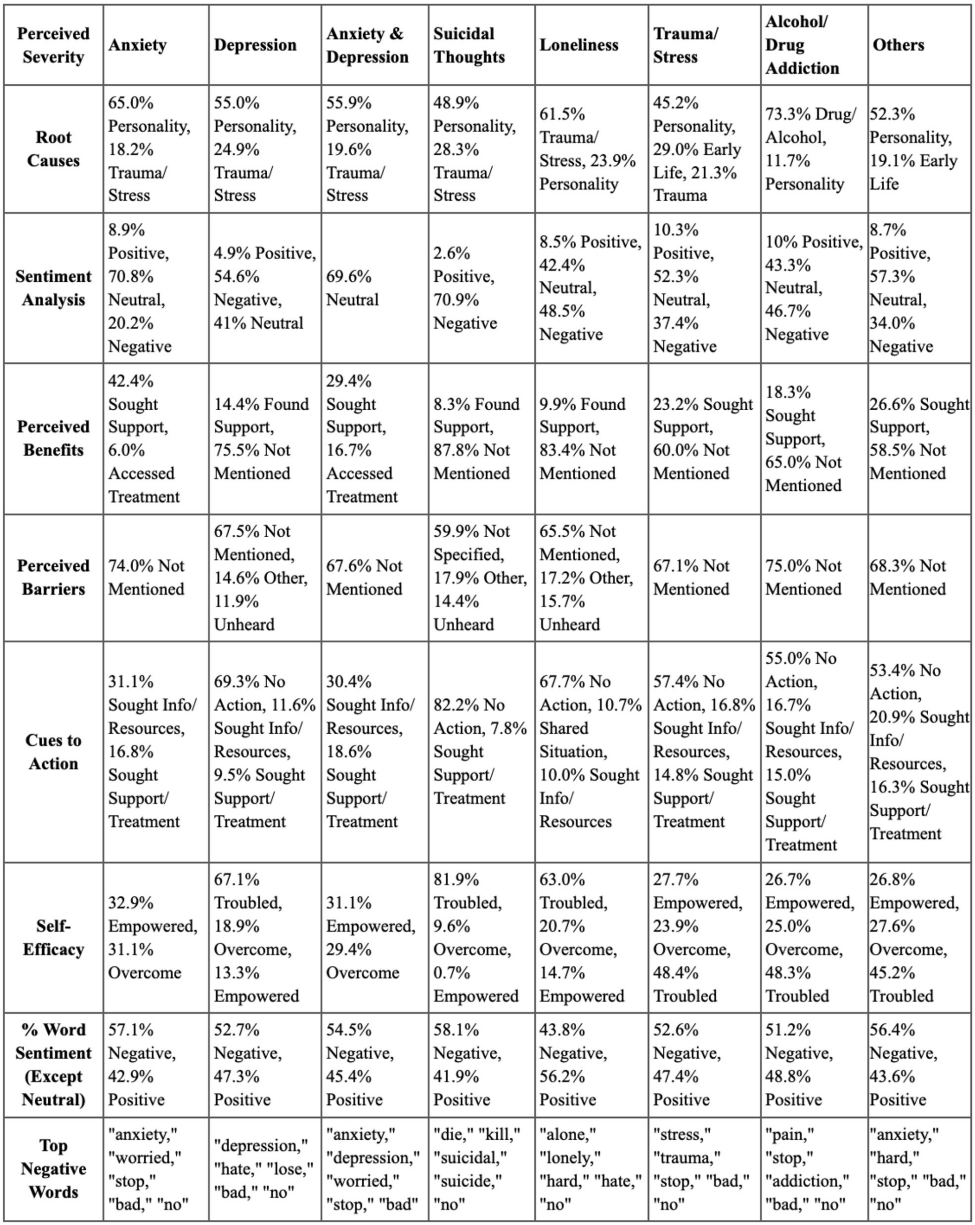
Overview of the key findings from the health belief model.

This section presents a consolidated overview of the key findings from the Health Belief Model Analysis and Emotional Content Analysis. The Figure 11 highlights the main components examined, such as perceived severity, susceptibility, self-efficacy, and emotional tone, summarizing the patterns and relationships observed across the dataset.

This integrated analysis offers a thorough understanding of health-related behaviors, perceptions, and emotional content within the context of mental health issues. Distinct patterns emerged in mental health issues: anxiety was influenced by personality traits and exhibited moderate negative sentiment. Depression showed high negative sentiment with significant inaction. Suicidal thoughts displayed the highest negative sentiment and extreme inaction, indicating a need for targeted interventions. Loneliness, associated with trauma, reflected balanced sentiment but substantial inaction.

Trauma and stress presented moderate negativity, while Alcohol and drug Addiction revealed high negative sentiment and significant inaction. The “others” category included various conditions with moderate negative sentiment and high inaction levels.

## 4 Discussion and future works

### 4.1 Discussion

This study aims to analyze health-related discussions through the lens of the HBM and to examine the emotional content within these interactions. Significant findings demonstrate that users tend to use more negative language in contexts with higher perceived severity.

Evaluating computational techniques for analyzing Reddit datasets within the HBM framework provides valuable insights into current methodologies for understanding mental health discussions online. This aligns with Boettcher (2021) findings on using Reddit data for studying mental health issues. The strong predictive performance for components like perceived susceptibility, perceived benefits, and self-efficacy (75%–84% accuracy) highlights the potential of these techniques. However, the low accuracy in predicting perceived severity (47%) reflects the challenges of multi-label classification, a common issue noted by Boettcher (2021).

The proposed hybrid approach, combining label prediction with keyword extraction, aligns with recent advancements in NLP and suggests that such methods could enhance accuracy in mental health text analysis. The superior performance of DistilBERT over other models reinforces the effectiveness of BERT-based architectures for this task, and integrating it with GPT-4o mini for text generation represents a promising direction.

Distinct patterns in mental health discussions reveal that anxiety correlates with moderate negative sentiment and self-efficacy, while depression shows high negative sentiment and inaction, indicating the need for empathetic interventions. suicidal thoughts exhibit the highest negativity and inaction, underscoring the urgency for targeted support. These findings resonate with Castilla-Puentes et al. (2022), highlighting the need for tailored interventions.

The analysis also provides valuable insights into mental health perceptions and behaviors. Our findings show that depression and anxiety were frequently discussed, reflecting high perceived severity, which aligns with Jones et al. (2015). However, the significant proportion of neutral sentiments suggests that many users may not fully recognize their susceptibility to mental health challenges.

The correlation between sentiment and self-efficacy supports Wong et al. (2021) emphasis on self-efficacy as a crucial predictor of health behaviors. Many users expressed feeling troubled in managing their mental health, indicating low self-efficacy. Additionally, the lack of mention of perceived benefits and barriers suggests a gap in understanding these factors, which could inform future interventions.

The low rate of reported action raises concerns about the effectiveness of cues to action in mental health contexts. A recent nationally representative survey study in Germany found that only 26% who had received a diagnosis of an anxiety disorder during lifetime ever were in contact with mental health services (Heinig et al., 2021). More than 70 % naturally never faced any barriers, as they did not even try to seek help. This discrepancy highlights the need for targeted cues to action in mental health especially with a focus on psycho-education. Studies focusing on help-seeking in depression revealed that being young or elderly, male and less educated increased the risk of not seeking support, as Magaard et al. (2017) did. The authors emphasize the role of primary care providers in facilitating communication.

Overall, while mental health conditions share common themes of negative sentiment and inaction, they also present unique characteristics that require multifaceted interventions addressing emotional and practical aspects. This nuanced understanding can lead to more effective and personalized support strategies. In this context it is important to understand that Reddit discussions are different from expert-driven medical forums. Jozani et al. (2025) added valuable results on dialogues in online health communities and emphasized the nature of informational and emotional support that complement each other. According to them, emotional support can even improve the effectiveness of information. Analyzing the emotional valence of mental health discussions on Reddit can inform experts about informational needs of patients and how to address them in an empathetic way.

### 4.2 Limitations

This research acknowledges several limitations. The quality of Reddit data varies, often containing noise that can skew analysis. Findings may not generalize to other platforms due to Reddit's unique culture. Inferring health beliefs involves subjective judgments that may overlook human complexities.

Additionally, human-labeled data can be biased, impacting model predictions. Analyzing emotional content presents challenges due to language nuances like sarcasm or the use of emotions. Temporal dynamics are not fully accounted for, as beliefs and emotions can shift over time. Computational methods may face processing power constraints, limiting the handling of large datasets. Ethical considerations about user privacy remain paramount, even with publicly accessible data.

Transformer models, while effective, struggle with rare words and domain adaptation. Enhancing domain adaptation techniques is necessary to capture the complexities of health-related concepts. Addressing these challenges will require methods for rare word handling and developing task-specific models. Improving interpretability and explainability in transformer models is vital for their practical application.

Another limitation of our study is that, while the broad 'root cause' categories may overlap as noted by Rani et al. (2024), our primary focus on the Health Belief Model means these categories were not the main focus; however, supplementary analyses, particularly of emotional content, offer more nuanced insights into perceived severity and suggest directions for future research and interventions.

Finally, demographic information, a central element of the Health Belief Model, could not be collected due to limitations of the Reddit dataset. Future research could incorporate demographic data to provide additional context and improve generalizability.

### 4.3 Future works

The computer-based approach presented here to analyze health-related discussions in Reddit data sets using the HBM can be applied to various contexts in future research designs. In addition to using data sets from other social media forums such as X or Instagram, topics that require a high degree of patient adherence appear to be particularly relevant. For example, previous research applying the HBM to successful smoking cessation found that perceived benefits of actions play a crucial role (Ravi et al., 2021). Analyzing online discussions with the methodology proposed in this work can inform experts about details of barriers and facilitators. Similar mechanisms were shown by applying the HBM to online discussions about human papillomavirus (HPV) vaccination (Li et al., 2022). Finally, the HBM has been applied to cancer prevention, where perceived susceptibility, benefits and cues to action were the most important elements of the model that were associated with screening behavior (Lau et al., 2020). In this context, previous results show that especially males appear to be less informed (Zafar et al., 2025). Gender-specific online information campaigns can directly address these needs.

Moreover, future research should focus on developing domain-specific models tailored to the HBM's complexities, including specialized vocabulary. Improved handling of rare words through advanced techniques like subword tokenization is essential for model robustness. Integrating temporal dynamics into analysis will provide deeper insights into evolving health beliefs and emotions. Scalability improvements using efficient transformer architectures can facilitate more extensive research. Exploring multimodal data sources, including images and videos, can enrich health-related content analysis.

Last not least, a more nuanced analysis of emotional content—assessing core emotions such as anger, fear, sadness, enjoyment, disgust, and surprise, as identified by Ekman (1992)—can further enhance understanding of user sentiments. Building on the approach of Jozani et al. (2025), who analyzed the emotional content of online health dialogues based on Ekman's framework, future work should address not only the content of posts, but as well the responses given by the respective communities. Addressing ethical considerations and biases in AI deployment is crucial for responsible research. Finally, comparing findings across various social media platforms will validate results and improve public health discussions.

## 5 Conclusion

This research highlights the effectiveness of computational techniques, particularly hybrid models like GPT-4o and DistilBERT, in analyzing mental health discussions on Reddit within the Health Belief Model framework. Our findings reveal distinct patterns in user sentiment and behavior, underscoring the need for tailored interventions to address various mental health conditions. Despite challenges such as low accuracy in predicting perceived severity and the complexities of multi-label classification, this study contributes to the understanding of how health beliefs shape public discourse. Future work should prioritize the development of domain-specific models, enhance data handling methods, and address ethical considerations to ensure responsible AI deployment in health research.

## Data Availability

The raw data supporting the conclusions of this article will be made available by the authors, without undue reservation.
